# Children and COVID- 19 infection: A case series of Iran

**DOI:** 10.22088/cjim.13.0.254

**Published:** 2022

**Authors:** Mohsen Mohammadi, Masood Kiani, Sanaz Mehrabani, Maryam Nikpour

**Affiliations:** 1 Non-Communicable Pediatric Disease Research Center, Health Research Institute, Babol University of Medical Sciences, Babol, Iran

**Keywords:** COVID- 19 infection, Children, Case series

## Abstract

**Background:**

World Health Organization (WHO) declared COVID -19 infection a global pandemic. Children have milder disease than adults but different aspects of disease in children are not fully understood.

**Case presentation:**

We describe 5 pediatric patients with COVID-19 that referred to Amirkola Children’s Hospital, Babol, Iran. The youngest patient was 4 years old and the oldest was 12 years old. Three patients were males. None of the patients had a history of contacts with symptomatic patients with COVID -19. The most common symptoms included fever, cough, anorexia, weakness and diarrhea. One patient had gastrointestinal symptoms without respiratory symptoms. All patients had elevated ESR and CRP. Three of them had lymphopenia. RT-PCR was positive in all patients. Management included supportive care, antibiotics, antiviral treatment and hydroxychloroquine. All patients were discharged with good condition.

**Conclusion:**

Children may have a variety of symptoms including respiratory or gastrointestinal symptoms. Mortality is rare in children and prognosis is better than the adults.

Respiratory infections caused by COVID-19 were first diagnosed in Wuhan, China, in December 2019, and "became one of the most important health problems in the world" (1). World Health Organization declared it as a pandemic (2).Coronaviruses are a family of viruses that can cause a wide range of illnesses, from cold to acute respiratory symptoms, and can cause death due to pneumonia and respiratory problems (3). COVID-19 can affect all ages, and children are no exception. The immune system of developing children and their response to the virus is different from adults (4). Dong et al. (4) in a study of 2143 children in China stated that 50% of these children with COVID-19 had mild respiratory or gastrointestinal symptoms such as fever, dry cough, sore throat, myalgia, nausea, vomiting, and diarrhea. Why children are less likely to develop the disease or have milder symptoms than adults is debatable, and may depend on host and guest factors. Angiotensin receptor is a transmitter of the COVID- 19 virus (5) and is likely to be involved in the pathogenesis of the virus. This receptor has less function in children than adults. Also in the Winter, children usually experience a variety of respiratory infections, so they are likely to have higher antibody levels to than adults.(4). In addition, children are cared at home and are less likely to be exposed to infected patients. Disease manifestations can be mild, moderate, severe or critical. Patients should be hospitalized if there are red flags including tachypnea, dyspnea, cyanosis, inability to eat or drink, decreased level of consciousness, signs of dehydration, fever more than 40˚C or lasting more than 3-5 days and spo2<93% in room air. Disease manifestations can be mild, moderate, severe or critical. 

Patients should be hospitalized if there are red flags including tachypnea, dyspnea, cyanosis, inability to eat or drink, decreased level of consciousness, signs of dehydration, fever more than 40˚C or lasting more than 3-5 days and spo2<93% in room air. Until today, no specific and standard treatment has been approved for patients with COVID- 19 and the current treatments used are based on experimental studies or preliminary results of clinical trials. Therapeutic evidence of adults is used in children due to the lack of sufficient information in children. All hospitalized patients receive supportive measures, including supplemental oxygen and other supportive therapies, and careful monitoring for symptom progression.

Medications such as atazanavir/ritonavir, lopinavir/ritonavir and INFB-1a are also recommended for hospitalized patients. Hydroxychloroquine was used at the beginning of the outbreak in hospitalized patients, but now is used in high risk patients with mild symptoms in the absence of contraindications. Antibiotics are prescribed if community-acquired pneumonia, sepsis and other infectious causes are suspected. Corticosteroids can be considered in some conditions, such as ARDS, HLH (hemophagocytic lymphohistiocytosis) or severe sepsis. In critical patients in which the previously mentioned drugs are not effective, according to infectious disease, pulmonary disease and anesthesiology specialists, high-dose corticosteroids, hemoperfusion and plasmapheresis can (6). Despite the global spread of the disease, the epidemiology and clinical pattern of COVID- 19 is still unknown, especially in children (4), and the risks associated with COVID -19 in children are not exactly known. although studies on clinical symptoms, laboratory results, and radiographic features and treatment of COVID -19 have been performed, but the focus of studies has been on adults, and in relation to the effect of COVID- 19 in children, very limited studies are available (7). Therefore, this study aimed to determine the clinical signs, laboratory results and radiographic characteristics of children with COVID -19 admitted at Amirkola Children’s Hospital, Babol, Iran. 

## Case presentations

**Figure1:**
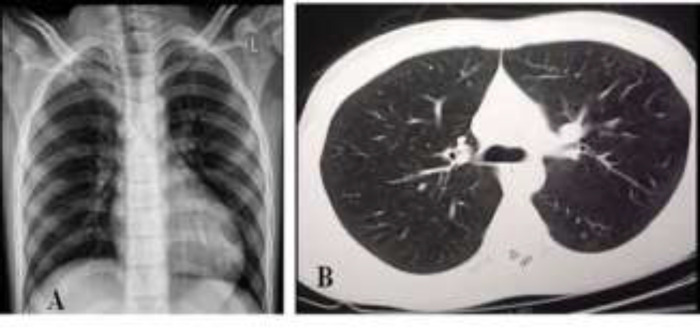
A, B: normal CXR and lung CT

**Figure 2:**
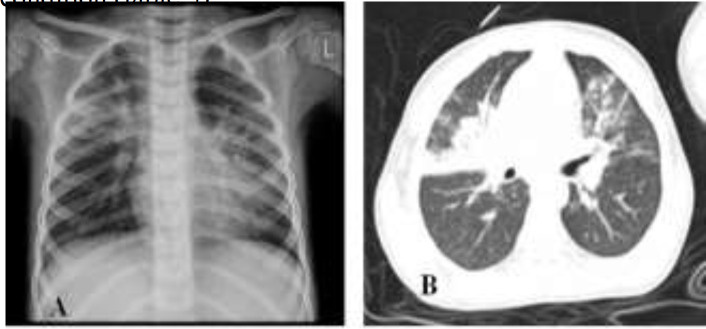
CXR (A) and lung CT (B) showed bilateral airspace consolidation and ground-glass opacity

**Figure 3:**
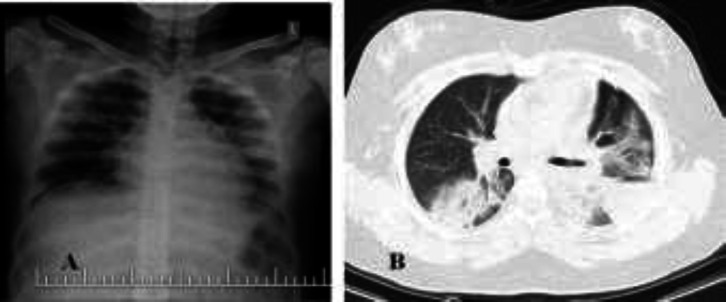
A, B CXR and lung CT findings: ground-glass opacity and consolidation in upper and lower lobes of left lung and right lower lobe

**Figure 4:**
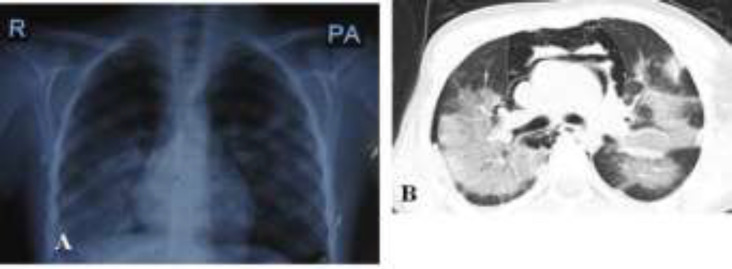
A, B CXR and lung CT findings: Multilober GGO and consolidation

**Figure 5:**
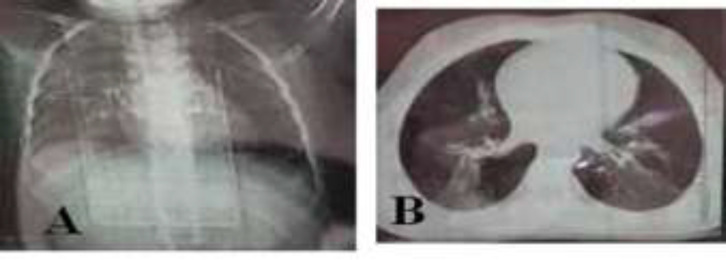
A, B CXR and lung CT findings: ground-glass opacity in both lungs

Clinical presentations, laboratory findings, radiologic features and treatments of cases are defined in tables1, 2, 3 and figures1-5 in brief, respectively.

**Table 1:** sign and symptoms of 5 patients with COVID 19

**Diarrhea**	**Anorexia**	**Weakness**	**Shortness of breath**	**Cough**	**Fever**	**Sex**	**Age(y)**	**Patient**
Y	Y	N	N	N	Y	m	12	1
N	Y	Y	N	Y	Y	m	4	2
Y	Y	Y	Y	Y	Y	f	12	3
N	Y	Y	Y	Y	Y	m	9	4
N	Y	Y	N	Y	Y	f	11	5

M: Male, F: Female, Y: Yes, N: No

**Table 2:** Laboratory findings of 5 patients with COVID 19

**RT-PCR**	**S/E**	**ESR (mm/h)**	**CRPmg/dl**	**PLT (103/l)**	**WBC (103/l)**	**Patient**
positive	WBC:30-35 RBC:20-25	50	60	189	4.6	1
positive	N	90	19	298	7.5	2
positive	N	16	42	191	5.3	3
positive	N	122	49	498	10	4
positive	N	35	67	375	7.1	5

N: No,,SE(stool exam),WBC:white blood cell,PLT:platelet ,ESR:erythrocyte sedimentation rate,CRP:c reactive protein

**Table 3:** Management of 5patients with COVID 19

**Mecanical ventilation**	**Methyl prednisolone**	**Oxygen**	**Hydroxychloroquin**	**Antiviral**	**Antibiotic**	**Patient**
N	N	N	N	N	N	1
N	N	N	Y	oseltamivir	Cefotaxime vancomycin	2
N	Y	Y	Y	Oseltamivir kaltra	Meropenem vancomycin	3
N	Y	Y	Y	Oseltamivir kaltra	Meropenem vancomycin	4
N	N	N	Y	N	Co amoxiclav	5

**Case 1: **A 12-year-old boy was referred to our emergency department. He had fever, anorexia, abdominal pain, vomiting and diarrhea that started two days ago. Diarrhea was not bloody. He did not have any respiratory symptoms (table 1). Except mild dehydration, other physical exams were normal. In his laboratory tests, he had elevated CRP and lymphopenia. Stool exam revealed leukocytes and red blood cells (table 2) but stool culture was negative (for shigella, Ecoli, salmonella, campylobacter and other bacteria and parasites). CXR and lung CT scan were normal (figure 1). RT-PCR was positive for COVID-19. The patient received supportive care, such as IV fluids and oral zinc and was discharged after three days with good general condition.

**Case 2: **A 4-year-old boy presented with cough, sore throat, weakness and anorexia for 10 days, and fever starting three days ago (table1). On physical examination, he had crackle in both lungs, no respiratory distress, and Spo2 was 98%. Other examinations were normal. He had no underlying disease. Chest-x-ray and lung CT scan showed extensive bilateral ground-glass opacity (GGO) and consolidation (figure 2). RT-PCR was positive for COVID-19. He was treated with antibiotics including cefotaxime and vancomycin plus hydroxychloroquine and oseltamivir. The patient was discharged from the hospital after 10 days with good general condition (table 3).

**Case 3: **A 12-year-old girl referred to emergency room with fever, cough, weakness, anorexia, diarrhea and shortness of breath that started two days ago (table 1). On physical examination, she had subcostal retraction, tachypnea, decreased breath sounds, crackle and end-expiratory wheezing. Spo2 was 88% in room air. The patient had a history of asthma. Laboratory test results included high CRP levels and lymphopenia. RT-PCR was positive for COVID-19 (table 2). CXR and CT scan showed bilateral GGO and consolidation (figure 3). She was treated with oxygen, antibiotics, hydroxychloroquine, oseltamivir and (lopinavir/ritonavir) (table3). Due to history of asthma, treatment with methylprednisolone, bronchodilators and inhaled corticosteroid (ICS) were performed. After 48 hours of hospitalization, the patient's respiratory distress improved and she was discharged from the hospital after 10 days with good condition. 

**Case 4: **A 9-year-old boy presented with fever, cough, weakness, anorexia, myalgia and shortness of breath (table1). Symptoms of the disease started one week ago. On physical examination, he had tachycardia, tachypnea, subcostal retraction and crackle in both lungs. Spo2 was 80%. He had history of asthma. ESR and CRP values were higher than normal. RT-PCR was positive for COVID-19 (table 2). CXR showed bilateral patchy opacity. 

Lung CT findings included bilateral GGO and consolidation (figure 4). The patient received supportive therapy, including oxygen, as well as antibiotics, hydroxychloroquine, oseltamivir, Kaletra, methylprednisolone, bronchodilators and ICS (table 3). After three days of treatment, the patient's respiratory distress and clinical condition improved. The patient was discharged after 9 days with good condition. 

**Case 5: **An 11-year-old girl presented with fever, cough, weakness and anorexia three days ago. She had no respiratory distress (table 1). Lung sounds and other physical examinations were normal. Lab tests included lymphopenia, high CRP and ESR. RT-PCR was positive for COVID-19 (table 2). Lung CT showed bilateral GGO (figure 5). She was treated on outpatient basis with co-Amoxiclav and hydroxychloroquine.

## Discussion

We described five Iranian children affected by COVID- 19. The most common signs and symptoms in our patients were fever, anorexia, weakness, cough and shortness of breath, respectively. Laboratory findings included lymphopenia, elevated ESR and CRP. All patients had positive RT-PCR test results for COVID-19. Additionally, the most important chest-x-ray and lung CT scan findings were ground-glass (GGO) opacity and consolidation.

The signs and symptoms in our patients were fever, anorexia, weakness, cough, myalgia and shortness of breath. Fever and anorexia were the most common. All of our patients experienced GI symptoms such as anorexia and two of them had diarrhea. Also, one case had gastrointestinal symptom) GI (symptoms without respiratory problems. The incidence of GI symptoms including anorexia, diarrhea, vomiting and abdominal pain in COVID -19 were 3-79% in literature. Fecal PCR testing was accurate as respiratory PCR detection (8). Two of our patients had severe disease (define as dyspnea, cyanosis or oxygen saturation blow 92%) (6) . Both patients with severe disease had underlying condition (asthma). In Wuhan children's hospital of 171 pediatric patients, three (1.8%) required intensive care and all of them had underlying disease (9). One patient had colitis. There are few case reports of colitis in COVID -19. One case report by Carvalho et al.(10) described a 71-year-old man with hemorrhagic colitis due to COVID -19. Wang et al. (8) found that of the 31 children with diarrhea 5.2% had leukocyte and 1.7% had occult blood in stool test results Among our patients, two had underlying disease including asthma. CDC report (11) reported the same finding. In their study of 345 pediatric patients in the US, 80 (23%) had at least one underlying condition in which the most common was chronic lung disease including asthma. In a study at Wuhan children's hospital of 171 children with COVID 19, three had underlying diseases including hydronephrosis, leukemia and intussusception (9). Patients' age ranged between 4-12 years. Of these, four were hospitalized and one treated as an outpatient basis. Three patients were males and two were females. In a study by Dong et al., 56.6% of patients were males (9). CDC report (11) of 2490 children with COVID -19 showed 57% were males. 

Laboratory findings in our cases included lymphopenia (3 cases), elevated ESR and CRP. No cases had thrombocytopenia. Liver and renal function tests were normal. In Rahimzadeh et al.’ study, abnormal laboratory findings in children with COVID-19 infection were leukopenia, lymphopenia, thrombocytopenia, elevated LDH, CPK, procalcitonin, ESR, and CRP (7). Chest-x-ray and lung CT scan findings in our patients included opacity ground-glass (GGO) and consolidation. One patient with only GI symptoms had normal CXR and lung CT scan. Radiologic features in COVID- 19 may be similar in children and adults and included GGO, consolidation, bronchial wall thickening and nodular opacities (7). Diagnosis of COVID -19 is based on clinical manifestations, history of contact and laboratory findings. In Dong et al.’s study (4), of 2143 pediatric patients, 34% had laboratory-confirmed diagnosis and the rest had clinically suspected disease. RT-PCR results were positive in all of our cases. We reported PCR positive patients in this study. Our patient treatment was supportive care including oxygen, IV fluids, hydroxychloroquine, oseltamivir, and antibiotics for bacterial superinfection. Two patients with severe diseases received lopinavir/ritonavir (Kaletra). For two patients with history of asthma in addition to treatments mentioned above, bronchodilators, inhaled and systemic corticosteroids were administered. Systemic corticosteroids have been used in severe disease; however, its benefits have not been determined. Therefore, corticosteroids should not be administered unless indicated for other reasons such as exacerbation of obstructive pulmonary disease or septic shock (12). It is also recommended that patients with asthma and chronic obstructive pulmonary disease (COPD) who are stable using inhaled corticosteroid, should continue their treatment at the time of COVID- 19 infection (13). None of our patients required admission to PICU and mechanical ventilation. There was no mortality among them and were discharged with good general condition. Ludvigsson et al. (9) reported that more than 90% of children with COVID- 19 infection, had asymptomatic, mild or moderate disease and about 5% had severe disease and less than 1% with critical disease. Mortality is very rare in pediatric patients. Of the 2572 pediatric cases in the US, three deaths were reported (11). This study has three limitations. First, it was a retrospective study that may have caused several systematic biases. Second, this study was conducted at a single center and third, case series study may be limited by the absence of standard interview questionnaires. 

In conclusion COVID 19 presentations may vary among pediatric patients. Some patients have only respiratory or GI symptoms and others have the combination of these symptoms. Children have milder disease than adults. Mortality is rare in children with COVID-19 infection, and these children usually have a good prognosis with appropriate treatment.
